# Evaluation of CSF albumin quotient in neuronal surface antibody-associated autoimmune encephalitis

**DOI:** 10.1186/s12987-022-00392-2

**Published:** 2022-11-23

**Authors:** Qi-Lun Lai, Meng-Ting Cai, Yang Zheng, Gao-Li Fang, Bing-Qing Du, Chun-Hong Shen, Jia-Jia Wang, Qin-Jie Weng, Yin-Xi Zhang

**Affiliations:** 1 Department of Neurology, Zhejiang Hospital, Hangzhou, 310013 China; 2 Department of Neurology, Second Affiliated Hospital, School of Medicine, Zhejiang University, Hangzhou, 310009 China; 3 Department of Neurology, First Affiliated Hospital of Zhejiang Chinese Medical University, Hangzhou, 310006 China; 4 Department of Neurology, Zhejiang Chinese Medicine and Western Medicine Integrated Hospital, Hangzhou, 310003 China; 5 Center for Drug Safety Evaluation and Research, College of Pharmaceutical Sciences, Zhejiang University, Hangzhou, 310058 China

**Keywords:** Neuronal surface antibody, Autoimmune encephalitis, Brain barriers, Cerebrospinal fluid / serum albumin quotient, Brain lesions, Prognosis

## Abstract

**Background and Objectives:**

Disruption of brain barriers is considered to be involved in the pathogenesis of neuronal surface antibody-associated autoimmune encephalitis (NSAE), but few studies have focused on their relationship. We aimed to explore the association between the integrity of brain barriers and clinical and paraclinical characteristics in patients with NSAE.

**Methods:**

This retrospective study consecutively recruited patients with NSAE. The cerebrospinal fluid (CSF) / serum albumin quotient (Qalb) was used to evaluate the function of brain barriers. The data on demographic information, clinical manifestations, magnetic resonance imaging (MRI), CSF findings and prognosis were collected and analyzed.

**Results:**

Of the 93 patients included, 33 (35.5%) patients were assigned to the elevated Qalb group and 60 (64.5%) patients to the normal Qalb group. Males and prodromal symptoms were more common in elevated Qalb group (both P < 0.05). The CSF white blood cell, protein, immunoglobulin G and albumin were significantly higher in elevated Qalb group (all P < 0.05). Patients with elevated Qalb were more likely to have brain lesions on MRI (60.6% versus 33.3%, P = 0.011). The modified Rankin Scale (mRS) scores at discharge and at last follow-up were significantly higher in patients with elevated Qalb than those with normal Qalb (both P < 0.05). After univariate and multivariate analyses, Qalb elevation (adjusted odds ratio = 3.96, 95% confidence interval = 1.15–13.59, P = 0.029) was demonstrated as the only independent risk factor for a poor prognosis.

**Discussion:**

Males, prodromal symptoms, brain lesions on MRI, CSF pleocytosis, and elevated CSF protein were more common in NSAE patients with increased Qalb. Qalb elevation was an independent prognostic indicator for a poor prognosis in NSAE.

**Supplementary Information:**

The online version contains supplementary material available at 10.1186/s12987-022-00392-2.

## Introduction

Autoimmune encephalitis (AE) is an expanding spectrum of non-infectious, inflammatory disorders of the central nervous system (CNS). Neuronal surface antibody-associated AE (NSAE), mediated by autoantibodies targeting against cell-surface and synaptic antigens, is the most common subgroup. The patients with NSAE typically manifest as acute / subacute onset of memory dysfunction, psychiatric symptoms, and seizures, with or without brain lesions on neuroimaging. Most of these patients respond well to immunotherapies in acute stage, and have a good clinical prognosis [1–3]. The exact pathogenesis of NSAE is still not clear. Studies have suggested that disruption of brain barriers might play an important role in the pathogenetic process of NSAE [4, 5]. It has been postulated that systemic lymphocytes and autoantibodies can enter the CNS through the damaged brain barriers, and these lymphocytes further differentiate into antibody-producing plasma cells and secrete specific autoantibodies directly against neurons [4, 5].

In clinical practice, the integrity of brain barriers cannot be evaluated directly by visualization. However, cerebrospinal fluid (CSF) / serum albumin quotient (Qalb) is currently recognized as the easiest and most reliable biomarker for evaluating the permeability of brain barriers, including blood–brain barrier and blood-CSF barrier [6], and has been used in a variety of neuroimmunological diseases [7], including multiple sclerosis (MS) and neuromyelitis optica spectrum disorders (NMOSD) [8, 9]. Nevertheless, few studies have focused on the relationship between Qalb and clinical and paraclinical features in AE patients. Therefore, the aim of the current study was to investigate the association between the Qalb and clinical and paraclinical characteristics, as well as prognosis in patients diagnosed with NSAE.

## Material and methods

### Patients

This retrospective study consecutively recruited patients with NSAE between January 2014 and December 2020 from Second Affiliated Hospital School of Medicine Zhejiang University. The inclusion criteria were as follows: (1) patients diagnosed as definite AE according to the 2016 Lancet Neurology criteria by Graus et al. [10]; (2) neuronal surface antibodies in the serum and / or CSF samples detected by cell-based assay; (3) serum and CSF albumin levels simultaneously obtained before immunotherapy. The exclusion criteria were as follows: (1) complicated with other neurological or psychiatric disorders (e.g. ischemic or hemorrhagic stroke, intracranial tumors, intracranial infection, toxic-metabolic encephalopathy, epilepsy, schizophrenia and others); (2) combination with other neural antibodies.

### Clinical data collection

In each enrolled participant, the following data were comprehensively collected: gender, onset age, disease duration (from disease onset to admission), prodromal presentations (including fever, headache, upper respiratory tract infection, nausea, vomiting, and diarrhea) [11], initial symptoms (including seizures, psychiatric symptoms, memory dysfunction, and others), requirement of intensive care unit (ICU) and treatment regimen. The modified Rankin Scale (mRS) was used to assess the disease severity at admission and the outcome at discharge (short-term) and at last follow-up (long-term). For the long-term clinical outcome in our study, mRS < 2 was defined as a good prognosis (without disability), while mRS ≥ 2 was defined as a poor prognosis (with disability).

### Acquirement and interpretation of neuroimaging

Brain magnetic resonance imaging (MRI) examinations were performed during the acute stage of NSAE using 1.5 T or 3.0 T scanner. Images were independently evaluated by two neurologists (QLL and YXZ). Brain MRI abnormalities were defined as new-onset brain lesions with abnormal signals on T1-weighted, T2-weighted, fluid attenuated inversion recovery (FLAIR), diffusion weighted or contrast-enhanced T1-weighted images.

### Blood and CSF analysis

Paired samples of serum and CSF were acquired simultaneously at acute stage of NSAE before immunotherapy. The CSF samples were tested for white blood cells, protein, albumin, immunoglobulin G (IgG), and intrathecal IgG synthesis index (IgG index). The albumin and IgG in serum and CSF samples were measured by standard nephelometric assay [12]. The parameter of Qalb was used to estimate the permeability of brain barriers [6]. As Qalb is age dependent, the individual age-related Qalb was defined as Qalb* = (4 + Age/15) × 10^–3^ [13]. In the current study, elevated Qalb (disruption of brain barriers) was defined as Qalb > Qalb* [14].

### Statistical analysis

The results were described as percentages, median and interquartile range (IQR). The Kolmogorov–Smirnov test was performed to evaluate the distribution of the variables. Univariate analyses were performed by Chi-square test or Fisher’s exact test for categorical data, and Mann–Whitney U test for continuous data in condition of non-normal distribution. Multivariate logistic regression analyses were taken after adjusting for different potential confounding factors to determine the risk factors of long-term prognosis. Odds ratio (OR) and 95% confidence interval (CI) were expressed for the risk estimate. P values < 0.05 were considered statistically significant. Statistical analyses were performed using SPSS (version 25.0) and R software (version 4.0.2).

## Results

### Demographic and clinical characteristics of the participants

Finally, 93 patients of NSAE were consecutively included, containing 56 with anti-N-methyl-D-aspartate receptor (NMDAR) encephalitis, 18 with anti-leucine-rich glioma-inactivated 1 encephalitis, 13 with anti-gamma-aminobutyric acid B receptor encephalitis, 3 with anti-contactin-associated protein-like 2 antibody-associated encephalitis, 2 with anti-alpha-amino-3-hydroxy-5-methyl-4-isoxazolepropionic acid receptor encephalitis, and 1 with anti-dipeptidyl-peptidase-like protein-6 encephalitis. Table 1 exhibited the demographic and clinical characteristics of the participants. Thirty (32.3%) patients were female. The median onset age and disease duration by the time of admission was 42.0 (IQR: 23.5, 57.5) years and 15.0 (IQR: 7.5, 30.0) days. Prodromal symptoms were present in 38 (40.9%) patients. The most common initial symptoms were seizures (65, 69.9%), psychiatric symptoms (43, 46.2%), and memory dysfunction (31, 33.3%). The mRS was used to assess disease severity of the cases, with a median score of 2.0 (IQR: 1.0, 3.0) at admission. Thirty-three (35.5%) patients with increased Qalb were assigned to the elevated Qalb group and the remaining 60 (64.5%) patients to the normal Qalb group.

**Table 1:** Comparison of demographic and clinical characteristics between normal and elevated Qalb groups of neuronal surface antibody-associated autoimmune encephalitis

	Total (n = 93)	Normal Qalb group (n = 60)	Elevated Qalb group (n = 33)	P value
Female, n (%)	30 (32.3)	24 (40.0)	6 (18.2)	0.031
Onset age, median (IQR)	42.0 (23.5, 57.5)	39.5 (23.0, 58.0)	47.0 (24.0, 56.5)	0.958
Presence of antibody, n (%)				
CSF	85 (91.4)	54 (90.0)	31 (93.9)	0.793
Serum	23 (24.7)	17 (28.3)	6 (18.2)	0.278
Antibody types, n (%)				
NMDAR	56 (60.2)	34 (56.7)	22 (66.7)	0.491
LGI1	18 (19.4)	14 (23.3)	4 (12.1)	
GABA_B_R	13 (14.0)	9 (15.0)	4 (12.1)	
Others	6 (6.5)	3 (5.1)	3 (9.1)	
Disease duration, days, median (IQR)	15.0 (7.5, 30.0)	14.0 (8.0, 30.0)	15.0 (6.0, 30.0)	0.885
Prodromal symptoms, n (%)	38 (40.9)	19 (31.7)	19 (57.6)	0.015
Concomitant tumors, n (%)	5 (5.4)	3 (5.0)	2 (6.1)	> 0.999
Initial symptoms, n (%)				
Seizures	65 (69.9)	46 (76.7)	19 (57.6)	0.055
Psychiatric symptoms	43 (46.2)	27 (45.0)	16 (48.5)	0.747
Memory dysfunction	31 (33.3)	21 (35.0)	10 (30.3)	0.646
Others	31 (33.3)	20 (33.3)	11 (33.3)	> 0.999
ICU requirement, n (%)	8 (8.6)	4 (6.7)	4 (12.1)	0.609
The mRS at admission, median (IQR)	2.0 (1.0, 3.0)	1.5 (1.0, 2.0)	2.0 (1.0, 3.0)	0.145

*CSF* cerebrospinal fluid, *GABA*_*B*_*R* gamma-aminobutyric acid B receptor, *ICU* intensive care unit, *IQR* interquartile range, *LGI1* leucine-rich glioma-inactivated 1, *mRS* modified Rankin Scale, *NMDAR* N-methyl-D-aspartate receptor, *Qalb* cerebrospinal fluid / serum albumin quotient

### Qalb and clinical manifestations

As shown in Table 1, there were more females in normal Qalb group of NSAE than those in elevated Qalb group (40% versus [vs.] 18.2%, P = 0.031). Prodromal symptoms were more common in elevated Qalb group than normal Qalb group (57.6% vs. 31.7%, P = 0.015). The median onset age, disease duration at admission, and antibody types were not significantly different between these groups (all P > 0.05). The initial symptoms, including seizures, psychiatric symptoms, memory dysfunction and other symptoms, were comparable among two subgroups (P > 0.05). As for the disease severity, there were tendencies that mRS scores at admission and ICU requirement during hospitalization were higher in elevated Qalb group than those in normal Qalb group, yet without statistical difference (Table 1).

Among the patients with anti-NMDAR encephalitis, females were more common in normal Qalb group than those in elevated Qalb group (55.9% vs. 22.7%, P = 0.014), with no significant difference in remaining characteristics, as displayed in Additional file 1: Table S1.

### Qalb and serum / CSF profiles

As shown in Table 2, the CSF white blood cell (WBC) was significantly higher in NSAE patients with elevated Qalb than those with normal Qalb group (11.0 [IQR: 4.0, 67.0] vs. 4.0 [IQR: 2.0, 8.0], P = 0.001). The CSF protein level was also significantly higher in elevated Qalb group than that in normal Qalb group (62.4 [IQR: 43.8, 102.2] vs. 30.8 [IQR: 26.6, 36.4], P < 0.001). The CSF IgG and albumin levels were significantly higher in elevated Qalb group than those in normal Qalb group (both P < 0.001). Serum IgG, albumin and IgG index were comparable among them (all P > 0.05).

**Table 2:** Comparison of paraclinical profiles between normal and elevated Qalb groups of neuronal surface antibody-associated autoimmune encephalitis

	Total (n = 93)	Normal Qalb group (n = 60)	Elevated Qalb group (n = 33)	P value
CSF analyses, median (IQR)				
WBC, × 10^6^/L	6.0 (2.0, 15.0)	4.0 (2.0, 8.0)	11.0 (4.0, 67.0)	0.001
Protein, mg/dL	36.2 (29.2, 50.1)	30.8 (26.6, 36.4)	62.4 (43.8, 102.2)	< 0.001
CSF IgG, mg/L	32.2 (23.9, 61.4)	27.3 (21.3, 34.0)	77.3 (53.6, 207.0)	< 0.001
CSF albumin, mg/L	207.0 (158.0, 336.0)	177.5 (132.3, 207.0)	426.0 (300.0, 676.5)	< 0.001
Serum IgG, g/L, median (IQR)	11.0 (9.6, 13.8)	11.1 (9.4, 13.9)	10.9 (9.7, 13.7)	0.866
Serum albumin, g/L, median (IQR)	37.7 (34.1, 42.1)	37.0 (34.1, 41.8)	38.5 (34.3, 42.9)	0.503
IgG index, median (IQR)	0.53 (0.46, 0.63)	0.52 (0.45, 0.58)	0.59 (0.44, 0.92)	0.065
Brain lesions, n (%)	40 (43.0)	20 (33.3)	20 (60.6)	0.011
Temporal lobe	32 (34.4)	17 (28.3)	15 (45.5)	0.096
Frontal lobe	17 (18.3)	7 (11.7)	10 (30.3)	0.026
Parietal lobe	8 (8.6)	2 (3.3)	6 (18.2)	0.040
Occipital lobe	4 (4.3)	2 (3.3)	2 (6.1)	0.931
Basal ganglion	6 (6.5)	1 (1.7)	5 (15.2)	0.036
Brainstem	5 (5.4)	0 (0.0)	5 (15.2)	0.009
Lesions with contrast	11/50 (22.0)	4/28 (14.3)	7/22 (31.8)	0.254

*CSF* cerebrospinal fluid, *IgG* immunoglobin *G, IQR* interquartile range, *Qalb* cerebrospinal fluid / serum albumin quotient, *WBC* white blood cell

For the patients with anti-NMDAR encephalitis, the CSF WBC, protein, IgG, and albumin levels were also significantly higher in elevated Qalb group than those in normal Qalb group (all P < 0.05). Serum IgG and albumin levels were comparable between these two groups (both P > 0.05). IgG index was higher in elevated Qalb group than that in normal Qalb group (P = 0.009) (Additional file 1: Table S2).

### Qalb and brain lesions on MRI

**Fig. 1:**
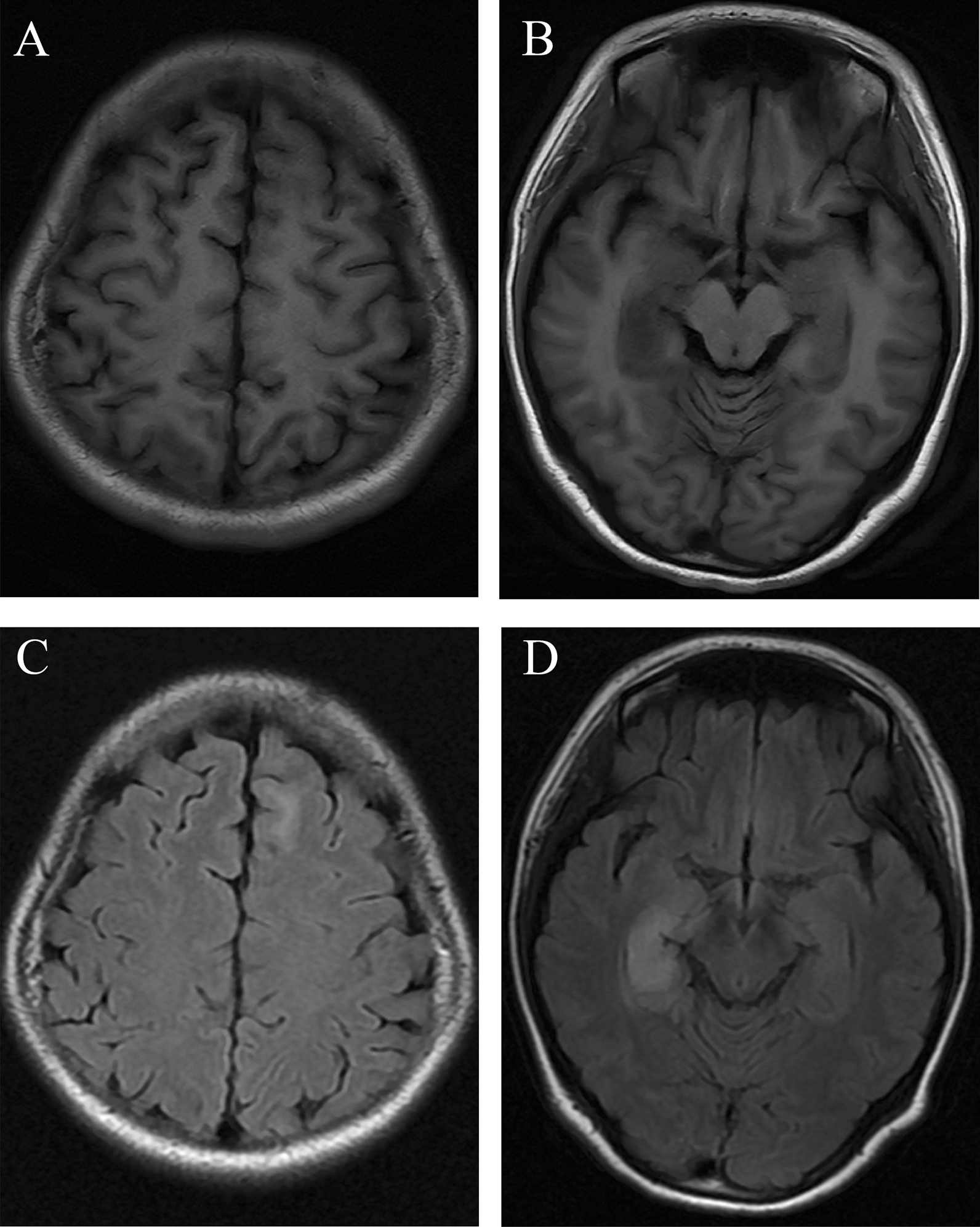
Brain magnetic resonance imaging findings of a young female patient with anti-leucine-rich glioma-inactivated 1 encephalitis. Axial T1-weighted imaging **A**, **B** showed hypointensities in left frontal lobe and right medial temporal lobe, with corresponding hyperintensities on axial fluid attenuated inversion recovery imaging **C**, **D**

Of the total 93 NSAE patients with MRI scan, brain lesions were found in 40 (43.0%) patients. Temporal lobe (32/93, 34.4%) and frontal lobe (17/93, 18.3%) were the mostly affected areas, as demonstrated in Fig. 1. The NSAE patients in elevated Qalb group were more likely to have brain lesions on MRI than those in normal Qalb group (60.6% vs. 33.3%, P = 0.011). As for the difference in lesion distribution, the frontal lobe, parietal lobe, basal ganglion and brainstem were more vulnerable to be involved in elevated Qalb group than those in normal Qalb group (all P < 0.05). The occurrence rates of lesions on temporal lobe, and occipital lobe were similar in normal and elevated Qalb groups (all P > 0.05). Fifty patients undertook brain MRI with contrast, and only 22.0% (11/50) of patients developed contrast-enhanced lesions. When comparing to normal Qalb group, the patients in elevated Qalb group tended to have contrast-enhanced lesions, but without statistical difference (31.8% vs. 14.3%, P = 0.254) (Table 2).

Similarly, anti-NMDAR encephalitis patients with elevated Qalb were more likely to have brain lesions than those with normal Qalb (68.2% vs. 26.5%, P = 0.002). The temporal lobe and brainstem lesions were more common in elevated Qalb group than those in normal Qalb group (both P < 0.05), while other brain regions were comparable (all P > 0.05) (Additional file 1: Table S2).

### Qalb and clinical outcome

**Fig. 2:**
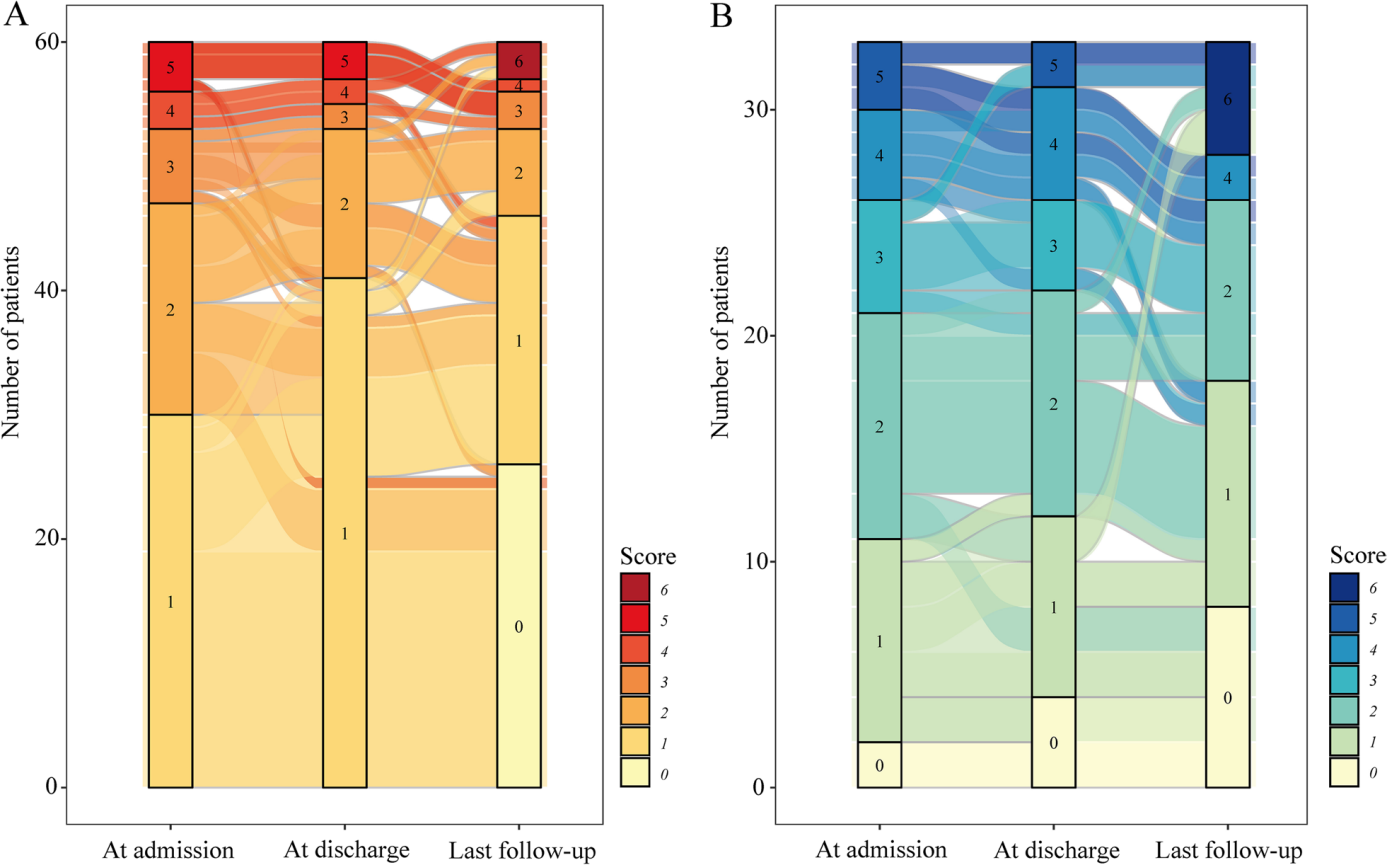
Sankey diagrams for visualizing the changes of modified Rankin Scale scores from admission to discharge and to last follow-up in patients with normal (**A**) and elevated (**B**) cerebrospinal fluid / serum albumin quotient. The flow lines between the three sidebars represent changes in individual scores. The height of the colored lodes and line width of the flow lines are the numbers of patients

During the hospitalization, most of the patients received first-line immunotherapies, including steroids (80/93, 86%) and intravenous immunoglobulin (74/93, 79.6%), which were similar in normal and elevated Qalb groups (both P > 0.05). The mRS scores at discharge were significantly higher in elevated Qalb group than those in normal Qalb group (2.0 [IQR: 1.0, 3.0] vs. 1.0 [IQR: 1.0, 2.0], P = 0.024). After a median follow-up time of 2.7 years, the median mRS score of the total cohort was 1.0 (IQR: 0.0, 2.0), and 64 (68.8%) patients achieved a good long-term prognosis (mRS < 2). The median mRS scores at last follow-up were still higher in elevated Qalb group than those in normal group (1.0 [IQR: 0.5, 2.0] vs. 1.0 [IQR: 0.0, 1.0], P = 0.021) (Table 3, Fig. 2). However, among the patients with anti-NMDAR encephalitis, the mRS at discharge and at last follow-up were not significantly different between the normal and elevated Qalb groups (both P > 0.05) (Additional file 1: Table S3).

**Table 3:** Comparison of clinical outcome between normal and elevated Qalb groups of neuronal surface antibody-associated autoimmune encephalitis

	Total (n = 93)	Normal Qalb group (n = 60)	Elevated Qalb group (n = 33)	P value
First-line immunotherapies, n (%)				
Steroids	80 (86.0)	54 (90.0)	26 (78.8)	0.238
IVIG	74 (79.6)	50 (83.3)	24 (72.7)	0.225
The mRS at discharge, median (IQR)	1.0 (1.0, 2.0)	1.0 (1.0, 2.0)	2.0 (1.0, 3.0)	0.024
Follow-up time, years, median (IQR)	2.7 (1.6, 4.6)	2.6 (1.6, 4.4)	3.8 (2.0, 5.0)	0.253
The mRS at last follow-up, median (IQR)	1.0 (0.0, 2.0)	1.0 (0.0, 1.0)	1.0 (0.5, 2.0)	0.021

*IQR* interquartile range, *IVIG* intravenous immunoglobulin, *mRS* modified Rankin Scale, *Qalb* cerebrospinal fluid / serum albumin quotient

To further explore the association between Qalb and long-term prognosis, the patients were divided into the good prognosis group (mRS < 2, n = 64) and the poor prognosis group (mRS ≥ 2, n = 29). For univariate analysis, as shown in the Additional file 1: Table S4, except for Qalb elevation and CSF protein level, all the other factors were not statistically different in the good and poor prognosis groups (all P > 0.05). To assess the significance of Qalb elevation as an independent risk factor, we further took multivariate analyses in view of different confounding factors. As exhibited in Additional file 1: Table S5, multivariate logistic regression models were performed after adjustment for increasing parameters, i.e., demographic, clinical and therapeutic factors. As intermediate factors, CSF profiles were not included in the multivariate analyses. As a result, the Qalb elevation was demonstrated as an independent risk factor for a poor prognosis (OR = 3.96, 95% CI = 1.15–13.59, P = 0.029).

## Discussion

To the best of our knowledge, this was the first study to explore the correlation between the Qalb elevation and clinical and paraclinical characteristics, as well as prognosis in patients with NSAE. Different from Yu et al.'s study [15], we expanded the population to various subtypes of NSAE, rather than anti-NMDAR encephalitis. In our study, we found that Qalb elevation was an independent prognostic indicator for a poor prognosis in NSAE. Though anti-NMDAR encephalitis and other subtypes of NSAE were different disease entities, disruption of brain barriers might be a common pathway of pathogenesis.

We showed that 35.5% of the patients developed disruption of brain barriers, as indicated by Qalb, which was previously reported in 27.4% ~ 29.3% of the patients with anti-NMDAR encephalitis [14, 15]. The brain barriers are essential to establish and maintain the microenvironment of brain that permits normal brain function. Hammer et al. found that the brain barriers-impaired APOE^−/−^ mice exhibited neuropsychiatric symptoms of anti-NMDAR encephalitis after injected with anti-NMDAR antibodies purified from the patient’s serum, while the wild-type mice with intact brain barriers were not behaviorally affected [16]. Our result indicated that prodromal symptoms were more common in patients with elevated Qalb. When triggered by precedingly infections, brain barriers can be damaged by direct injury of microorganisms and subsequent immunological response. Lymphocytes and autoantibodies targeting neuronal surface antigens enter the CNS through the disrupted brain barriers, contributing to the pathogenesis of NSAE [4, 5].

We discovered that females were more common in NSAE patients with normal Qalb, indicating that female might be a protective factor for the integrity of brain barriers. May et al. identified a novel protective mechanism of estrogen upon the brain barriers, showing that estrogen could both enhance inter-endothelial cell tight junction function and limit lymphocyte transmigration, via regulating downstream protein annexin A1 [17]. In a study of experimental autoimmune encephalomyelitis animal model, male mice showed greater disease severity and more pronounced neurodegeneration than female mice [18]. In a latest MS cohort enrolling 10770 patients, female patients exhibited a slower annualised increase in expanded disability status scale scores. These studies supported the protective role of female in the permeability of brain barriers [19].

Our result suggested that the CSF WBC, albumin, IgG, and protein levels were higher in elevated Qalb group of NSAE patients, while IgG index reflecting intrathecal IgG synthesis was similar in both groups. There are two sources of IgG in CSF: one is synthesized within the CNS by antibody-producing cells, and the other is from serum through the brain barriers, which is synthesized in the liver [5]. Under normal circumstances, the brain barriers prevent neurotoxic plasma components, blood cells, and pathogens from crossing the brain barriers and entering the CNS [20]. When the brain barriers are disrupted, serum albumin and IgG can enter the CNS and then increase the amount of protein in the CSF. Similarly, serum WBC, mainly lymphocytes, are able to get into CNS through impaired brain barriers and participate in the pathogenic process of NSAE along with autoantibodies [4].

Brain lesions on MRI were found in 43% of our patients, and temporal and frontal lobes were the mostly affected areas. Our result was consistent with the reported prevalence of abnormal brain MRI, which ranged from 30.0% to 73.0% in two multicenter studies, mainly involving the medial temporal lobe [2, 21]. As we indicated, brain lesions were more likely to be detected in patients with elevated Qalb, and frontal lobe, parietal lobe, basal ganglion and brainstem were more vulnerable to be involved among these patients. However, the intracranial lesions detected on MRI did not differentiate between the normal and damaged brain barriers groups in another study containing 73 patients with anti-NMDAR encephalitis [15]. Contrast-enhanced lesion on MRI was supposed to be associated with disrupted brain barriers in various intracranial diseases [22]. But the occurrence of contrast-enhanced lesions was not significantly higher in our patients with elevated Qalb than those with normal Qalb, probably due to the limited number of cases having contrast-enhanced brain scans. Hence, the associations between disruption of brain barriers and presence of brain lesions, and contrast-enhanced lesions, need to be further clarified in larger cohorts.

In our study, most of the patients achieved favorable long-term functional outcome, which was similar to reported studies [1–3]. The mRS scores at discharge and at last follow-up were significantly higher in elevated Qalb group than those in normal Qalb group. Furthermore, multivariate logistic regression analysis indicated that Qalb elevation (adjusted OR = 3.96, 95% CI = 1.15–13.59) was an independent risk factor for a poor prognosis. These results suggested that patients with intact brain barriers might be more sensitive to first-line immunotherapies and have better short- and long-term prognosis. When the brain barriers are damaged, more immune cells and autoantibodies in the blood enter the CNS, and possibly prompt a more robust immune response to the brain, consequently leading to a poor prognosis. Thus, Qalb elevation might be a potential prognostic indicator in patients with NSAE.

In contrast to prognosis, our result indicated that the disease severity at admission measured by mRS were not significantly different between the normal and elevated Qalb groups. However, in another study, anti-NMDAR encephalitis patients with impaired brain barriers were more likely to have a higher disease severity before immunotherapy [15]. With regard to other inflammatory diseases of CNS, disruption of brain barriers integrity was shown to be associated with higher disease severity in patients with NMOSD, but not in patients with MS [8, 9]. Hence, the correlation between the initial disease severity and Qalb elevation needs to be further determined.

In the sub-set of anti-NMDAR encephalitis, most of the clinical and paraclinical features were consistent with overall NSAE. Exceptionally, the presence of prodromal symptoms was similar between the normal and elevated Qalb groups. Additionally, the short- and long-term outcomes were comparable between these groups, which was also different from the short-term prognosis of Yu et al.'s study [15]. The difference might be due to the limited number of patients with anti-NMDAR encephalitis included, and larger sample sizes are needed to clarify the relevance.

There were several limitations in our study. Firstly, there might be existence of information bias, as the present study was a retrospective single-centre cohort study. Secondly, a certain of patients without simultaneous serum and CSF albumin testing were excluded from our analysis, which might lead to selection bias. Thirdly, our study included limited cases of different antibody subtypes, thus subgroup analysis was only conducted in anti-NMDAR encephalitis. Therefore, prospective multi-center studies with larger number of patients and longer follow-up should be carried out to further validate our results.

## Conclusion

Our study revealed that Qalb elevation was present in over one-third of the patients with NSAE. Male and prodromal symptoms were more common with elevated Qalb. Higher prevalence of CSF pleocytosis, elevated CSF protein, and brain lesions on MRI were observed in patients with increased Qalb. Furthermore, Qalb elevation was an independent prognostic indicator for a poor prognosis in patients with NSAE.

## Supplementary Information

**Additional file 1: Table S1.** Comparison of demographic and clinical characteristics between normal and elevated Qalb groups of anti-NMDAR encephalitis. **Table S2.** Comparison of paraclinical profiles between normal and elevated Qalb groups of anti-NMDAR encephalitis. **Table S3.** Comparison of clinical outcome between normal and elevated Qalb groups of anti-NMDAR encephalitis. **Table S4.** Comparison of clinical and paraclinical characteristics between good and poor prognosis groups of neuronal surface antibody-associated autoimmune encephalitis. **Table S5.** Multivariate logistic regression analyses for a poor long-term prognosis of neuronal surface antibody-associated autoimmune encephalitis

## Data Availability

Anonymized data not published within this article will be made available upon reasonable request from any qualified investigator within 5 years after publication.
