# Supplementary material for: Evaluation of CSF albumin quotient in neuronal surface antibody-associated autoimmune encephalitis
Source: Fluids Barriers CNS. 2022 Nov 23;19:93. doi: 10.1186/s12987-022-00392-2 (PMC9685895; doi:10.1186/s12987-022-00392-2)
Supplement: Supplementary file 1 — Additional file 1: Table S1. Comparison of demographic and clinical characteristics between normal and elevated Qalb groups of anti-NMDAR encephalitis. Table S2. Comparison of paraclinical profiles between normal and elevated Qalb groups of anti-NMDAR encephalitis. Table S3. Comparison of clinical outcome between normal and elevated Qalb groups of anti-NMDAR encephalitis. Table S4. Comparison of clinical and paraclinical characteristics between good and poor prognosis groups of neuronal surface antibody-associated autoimmune encephalitis. Table S5. Multivariate logistic regression analyses for a poor long-term prognosis of neuronal surface antibody-associated autoimmune encephalitis [file 12987_2022_392_MOESM1_ESM.docx]

Additional file 1: **Table S1. Comparison of demographic and clinical characteristics between normal and elevated Qalb groups of anti-NMDAR encephalitis**

|  | Total (n=56) | Normal Qalb group  (n=34) | Elevated Qalb group  (n=22) | P value |
| --- | --- | --- | --- | --- |
| Female, n (%) | 24 (42.9) | 19 (55.9) | 5 (22.7) | 0.014 |
| Onset age, median (IQR) | 24.5 (21.3, 41.5) | 24.5 (20.8, 34.5) | 24.5 (22.3, 54.0) | 0.406 |
| Presence of antibody, n (%) | | | | |
| CSF | 54 (96.4) | 32 (94.1) | 22 (100.0) | 0.514 |
| Serum | 14 (25.0) | 10 (29.4) | 4 (18.2) | 0.527 |
| Disease duration, days, median (IQR) | 14.5 (7.3, 30.0) | 14.0 (7.0, 30.0) | 15.0 (9.3, 30.0) | 0.538 |
| Prodromal symptoms, n (%) | 33 (58.9) | 17 (50.0) | 16 (72.7) | 0.091 |
| Concomitant tumors, n (%) | 1 (1.8) | 1 (2.9) | 0 (0.0) | >0.999 |
| Initial symptoms, n (%) | | | | |
| Seizures | 36 (64.3) | 24 (70.6) | 12 (54.5) | 0.221 |
| Psychiatric symptoms | 29 (51.8) | 18 (52.9) | 11 (50.0) | 0.830 |
| Memory dysfunction | 12 (21.4) | 8 (23.5) | 4 (18.2) | 0.886 |
| Others | 25 (64.3) | 15 (44.1) | 10 (45.5) | 0.922 |
| ICU requirement, n (%) | 8 (14.3) | 4 (11.8) | 4 (18.2) | 0.780 |
| The mRS at admission, median (IQR) | 2.0 (1.0, 3.0) | 1.0 (1.0, 2.3) | 2.0 (1.0, 3.3) | 0.582 |

Abbreviations: CSF, cerebrospinal fluid; ICU, intensive care unit; IQR, interquartile range; mRS, modified Rankin Scale; NMDAR, N-methyl-D-aspartate receptor; Qalb, cerebrospinal fluid / serum albumin quotient.

Additional file 1: **Table S2. Comparison of paraclinical profiles between normal and elevated Qalb groups of anti-NMDAR encephalitis**

|  | Total (n=56) | Normal Qalb group  (n=34) | Elevated Qalb group  (n=22) | P value |
| --- | --- | --- | --- | --- |
| CSF analyses, median (IQR) | | | | |
| WBC, ×10^6^/L | 6.5 (4.0, 40.0) | 5.0 (2.0, 13.5) | 23.0 (4.0, 107.5) | 0.020 |
| Protein, mg/dL | 35.4 (28.1, 49.5) | 29.2 (26.4, 33.2) | 59.7 (40.6, 83.4) | <0.001 |
| CSF IgG, mg/L | 32.1 (22.8, 64.5) | 25.4 (20.2, 32.0) | 82.0 (54.6, 134.3) | <0.001 |
| CSF albumin, mg/L | 206.5 (145.0, 340.0) | 158.5 (126.5, 194.0) | 415.5 (264.3, 570.5) | <0.001 |
| Serum IgG, g/L, median (IQR) | 11.0 (8.9, 13.5) | 11.0 (10.1, 14.4) | 10.7 (8.4, 12.5) | 0.302 |
| Serum albumin, g/L, median (IQR) | 38.7 (34.7, 43.0) | 37.8 (34.6, 42.5) | 39.8 (34.8, 45.6) | 0.455 |
| IgG index, median (IQR) | 0.56 (0.47, 0.66) | 0.54 (0.46, 0.60) | 0.64 (0.50, 1.05) | 0.009 |
| Brain lesions, n (%) | 24 (42.9) | 9 (26.5) | 15 (68.2) | 0.002 |
| Temporal lobe | 16 (28.1) | 6 (17.6) | 10 (45.5) | 0.024 |
| Frontal lobe | 12 (21.1) | 5 (14.7) | 7 (31.8) | 0.234 |
| Parietal lobe | 7 (12.3) | 2 (5.9) | 5 (22.7) | 0.148 |
| Occipital lobe | 3 (5.3) | 2 (5.9) | 1 (4.5) | >0.999 |
| Basal ganglion | 5 (8.8) | 1 (2.9) | 4 (18.2) | 0.141 |
| Brainstem | 5 (8.8) | 0 (0.0) | 5 (22.7) | 0.015 |
| Lesions with contrast | 7/30 (23.3) | 1/15 (6.7) | 6/15 (0.40) | 0.084 |

Abbreviations: CSF, cerebrospinal fluid; IgG, immunoglobin G; IQR, interquartile range; NMDAR, N-methyl-D-aspartate receptor; Qalb, cerebrospinal fluid / serum albumin quotient; WBC, white blood cell.

Additional file 1: **Table S3. Comparison of clinical outcome between normal and elevated Qalb groups of anti-NMDAR encephalitis**

|  | Total (n=56) | Normal Qalb group  (n=34) | Elevated Qalb group  (n=22) | P value |
| --- | --- | --- | --- | --- |
| First-line immunotherapies, n (%) | | | | |
| Steroids | 49 (87.5) | 30 (88.2) | 19 (86.4) | >0.999 |
| IVIG | 41 (73.2) | 26 (76.5) | 15 (68.2) | 0.494 |
| The mRS at discharge, median (IQR) | 1.0 (1.0, 2.0) | 1.0 (1.0, 2.0) | 2.0 (1.0, 3.3) | 0.525 |
| Follow-up time, years, median (IQR) | 3.1 (1.6, 5.0) | 2.9 (1.8, 5.4) | 3.9 (0.7, 4.9) | 0.913 |
| The mRS at last follow up, median (IQR) | 1.0 (0.0, 2.0) | 1.0 (0.0, 1.3) | 1.0 (0.0, 2.0) | 0.320 |

Abbreviations: IQR, interquartile range; IVIG, intravenous immunoglobulin; mRS, modified Rankin Scale; NMDAR, N-methyl-D-aspartate receptor; Qalb, cerebrospinal fluid / serum albumin quotient.

Additional file 1: **Table S4. Comparison of clinical and paraclinical characteristics between good and poor prognosis groups of neuronal surface antibody-associated autoimmune encephalitis**

|  | The mRS < 2 group (n=64) | The mRS ≥ 2 group (n=29) | P value |
| --- | --- | --- | --- |
| Female, n (%) | 21 (32.8) | 9 (31.0) | 0.865 |
| Onset age, median (IQR) | 34.5 (24.0, 57.0) | 54.0 (23.0, 64.0) | 0.250 |
| Presence of antibody, n (%) | | | |
| Serum | 14 (21.9) | 9 (31.0) | 0.343 |
| CSF | 59 (92.2) | 26 (89.7) | 0.997 |
| Antibody types, n (%) | | | |
| NMDAR | 41 (64.1) | 15 (51.7) | 0.144 |
| LGI1 | 14 (21.9) | 4 (13.8) |  |
| GABA_B_R | 6 (9.4) | 7 (24.1) |  |
| Others | 3 (4.7) | 3 (10.3) |  |
| Disease duration, days, median (IQR) | 15.5 (10.0, 30.0) | 13.0 (5.0, 27.5) | 0.415 |
| Prodromal symptoms, n (%) | 28 (43.8) | 10 (34.5) | 0.400 |
| Concomitant tumors, n (%) | 2 (3.1) | 3 (10.3) | 0.350 |
| Initial symptoms, n (%) | | | |
| Seizures | 46 (71.9) | 19 (65.5) | 0.536 |
| Psychiatric symptoms | 28 (43.8) | 15 (51.7) | 0.475 |
| Memory dysfunction | 23 (35.9) | 8 (27.6) | 0.429 |
| Others | 24 (37.5) | 7 (24.1) | 0.205 |
| Qalb elevation, n (%) | 18 (28.1) | 15 (51.7) | 0.028 |
| CSF analyses, median (IQR) |  |  |  |
| WBC, ×10^6^/L | 6.0 (2.0, 25.5) | 4.0 (2.0, 11.5) | 0.342 |
| Protein, mg/dL | 34.1 (28.2, 42.0) | 41.7 (31.7, 65.9) | 0.038 |
| CSF IgG, mg/L | 31.6 (21.9, 54.6) | 37.9 (27.8, 137.0) | 0.064 |
| CSF albumin, mg/L | 204.5 (158.0, 273.0) | 247.0 (156.0, 386.0) | 0.199 |
| Brain lesions, n (%) | 28 (43.8) | 12 (41.4) | 0.831 |
| First-line immunotherapies, n (%) | | | |
| Steroids | 58 (90.6) | 22 (75.9) | 0.114 |
| IVIG | 50 (78.1) | 24 (82.8) | 0.608 |
| Time of follow-up, years, median (IQR) | 2.5 (1.3, 4.4) | 3.8 (1.5, 5.7) | 0.172 |

Abbreviations: CSF, cerebrospinal fluid; GABA_B_R, gamma-aminobutyric acid B receptor; IgG, immunoglobulin G; IQR, interquartile range; IVIG, intravenous immunoglobulin; LGI1, leucine-rich glioma-inactivated 1; mRS, modified Rankin Scale; NMDAR, N-methyl-D-aspartate receptor; Qalb, cerebrospinal fluid / serum albumin quotient; WBC, white blood cell.

Additional file 1: **Table S5. Multivariate logistic regression analyses for a poor long-term prognosis of neuronal surface antibody-associated autoimmune encephalitis.**

| Models | Variables | OR | 95% CI | P value |
| --- | --- | --- | --- | --- |
| Model 1 | Qalb elevation | 3.20 | 1.21-8.45 | 0.019 |
|  | Female | 0.55 | 0.18-1.71 | 0.301 |
|  | Onset age | 1.02 | 1.00-1.05 | 0.094 |
| Model 2 | Qalb elevation | 3.69 | 1.25-10.93 | 0.018 |
|  | Female | 1.89 | 0.56-6.39 | 0.307 |
|  | Onset age | 1.02 | 0.99-1.05 | 0.233 |
|  | Disease duration | 1.00 | 0.99-1.02 | 0.458 |
|  | Prodromal symptoms | 0.44 | 0.14-1.38 | 0.158 |
|  | Concomitant tumors | 3.20 | 0.43-23.85 | 0.256 |
|  | Initial symptoms | | | |
|  | Seizures | 0.70 | 0.24-2.11 | 0.532 |
|  | Psychiatric symptoms | 1.70 | 0.63-4.61 | 0.296 |
|  | Memory dysfunction | 0.33 | 0.09-1.18 | 0.089 |
|  | Others | 0.54 | 0.18-1.65 | 0.279 |
| Model 3 | Qalb elevation | 3.96 | 1.15-13.59 | 0.029 |
|  | Female | 3.15 | 0.79-12.67 | 0.105 |
|  | Onset age | 1.02 | 0.98-1.07 | 0.29 |
|  | Disease duration | 1.01 | 0.99-1.02 | 0.202 |
|  | Prodromal symptoms | 0.47 | 0.11-1.97 | 0.300 |
|  | Concomitant tumors | 1.20 | 0.13-11.40 | 0.877 |
|  | Initial symptoms | | | |
|  | Seizures | 0.47 | 0.11-1.99 | 0.302 |
|  | Psychiatric symptoms | 2.19 | 0.63-7.53 | 0.216 |
|  | Memory dysfunction | 0.36 | 0.08-1.73 | 0.204 |
|  | Others | 0.44 | 0.12-1.60 | 0.210 |
|  | Presence of antibody | | | |
|  | Serum | 3.96 | 0.76-20.75 | 0.104 |
|  | CSF | 0.81 | 0.06-11.01 | 0.872 |
|  | Antibody types | | | |
|  | NMDAR | refence |  |  |
|  | LGI1 | 0.39 | 0.03-4.29 | 0.437 |
|  | GABA_B_R | 0.19 | 0.01-2.61 | 0.212 |
|  | Others | 1.05 | 0.07-16.11 | 0.97 |
|  | Brain lesions | 1.33 | 0.38-4.70 | 0.655 |
|  | First-line immunotherapies | | | |
|  | Steroids | 0.46 | 0.08-2.48 | 0.365 |
|  | IVIG | 0.95 | 0.22-4.09 | 0.944 |
|  | Time of follow-up | 1.22 | 0.88-1.71 | 0.231 |

Abbreviations: CI, confidence interval; CSF, cerebrospinal fluid; GABA_B_R, gamma-aminobutyric acid B receptor; IVIG, intravenous immunoglobulin; LGI1, leucine-rich glioma-inactivated 1; NMDAR, N-methyl-D-aspartate receptor; OR, odds ratio; Qalb, cerebrospinal fluid / serum albumin quotient.
